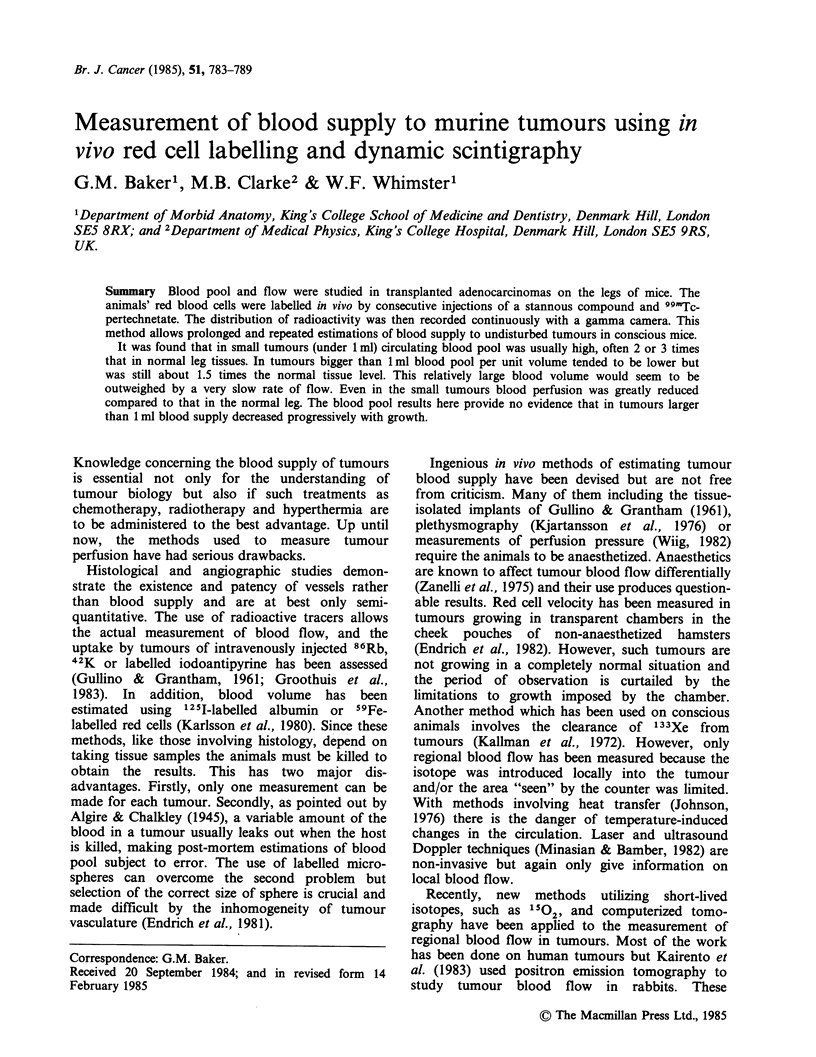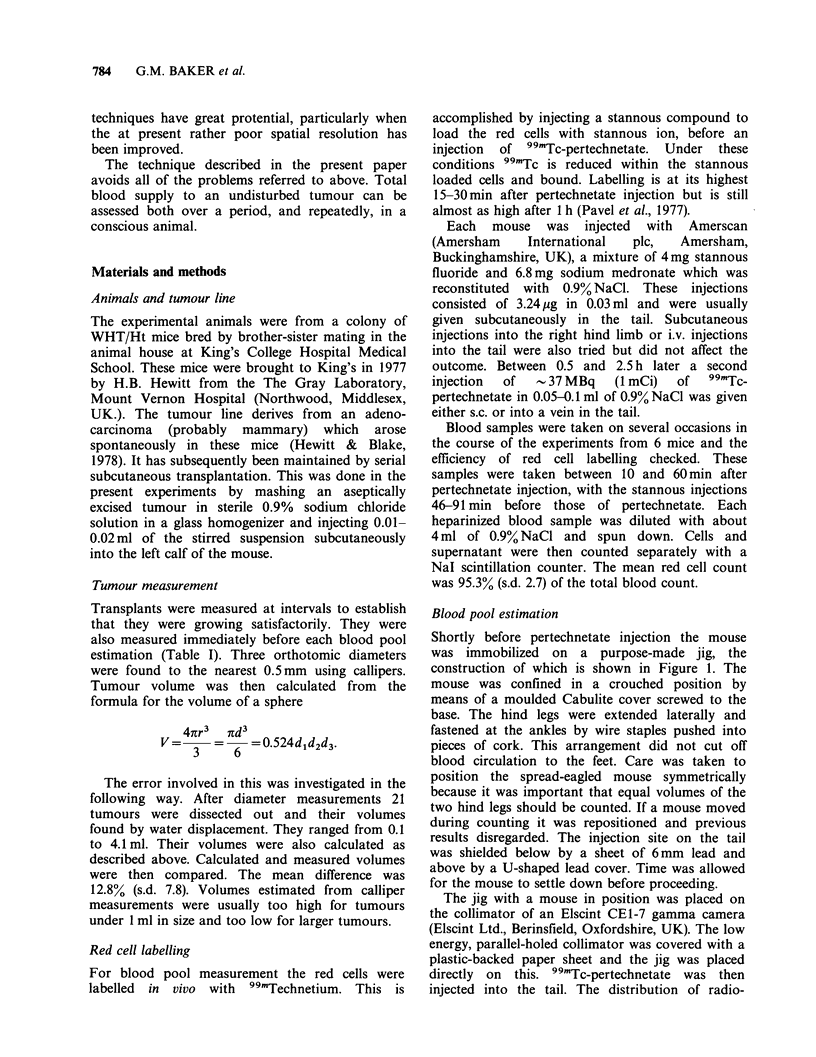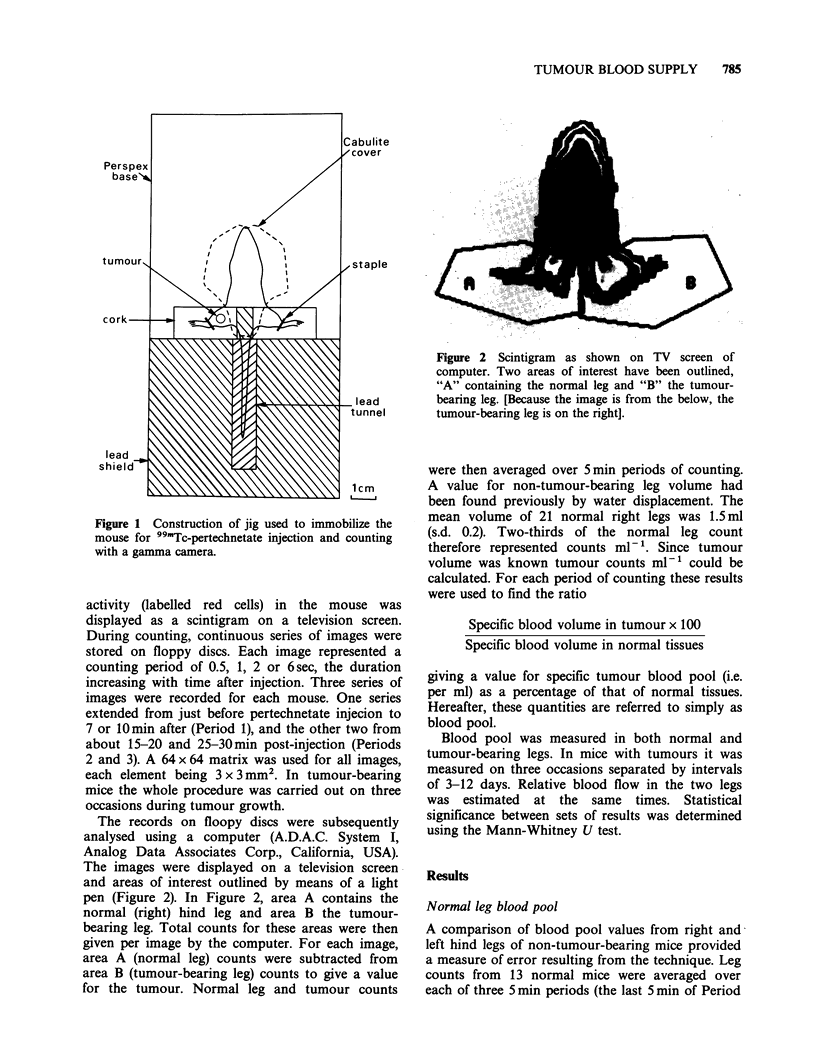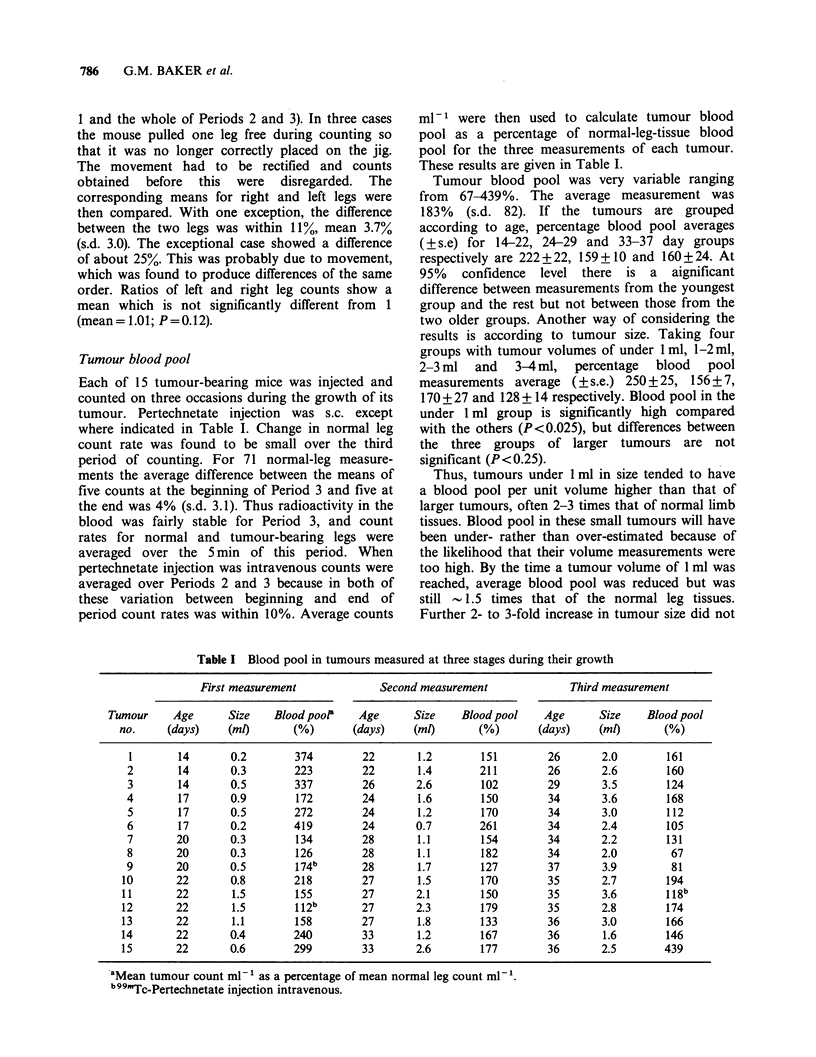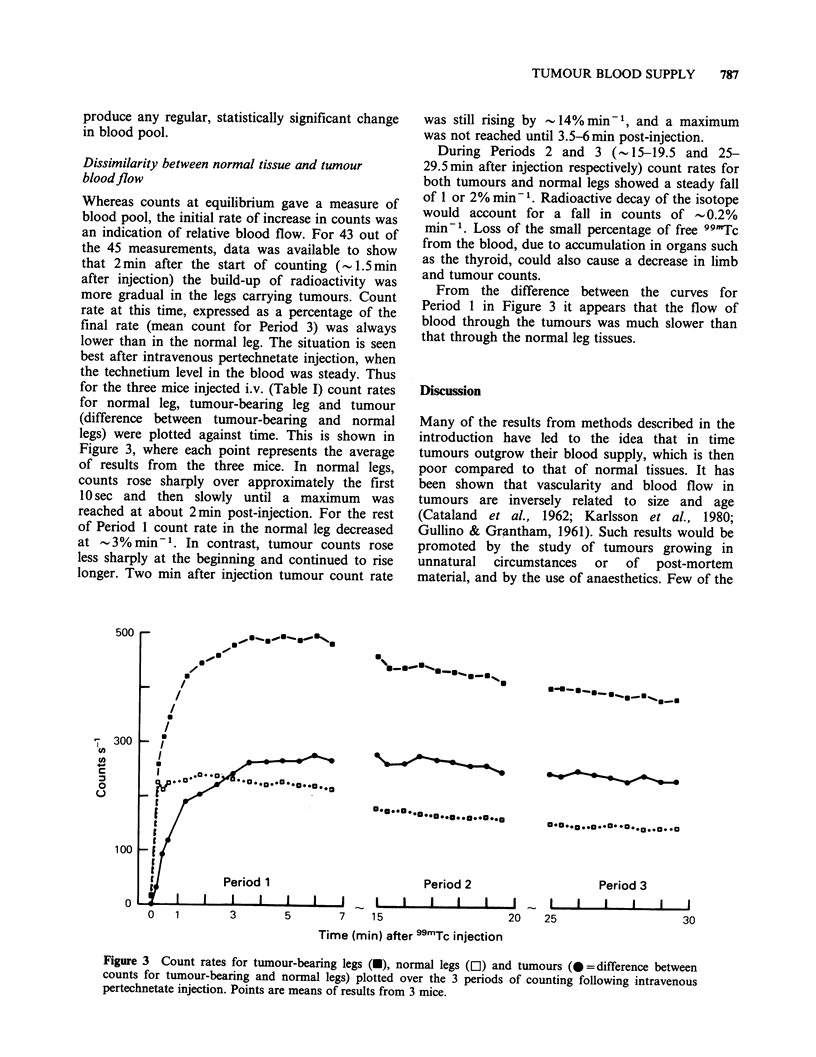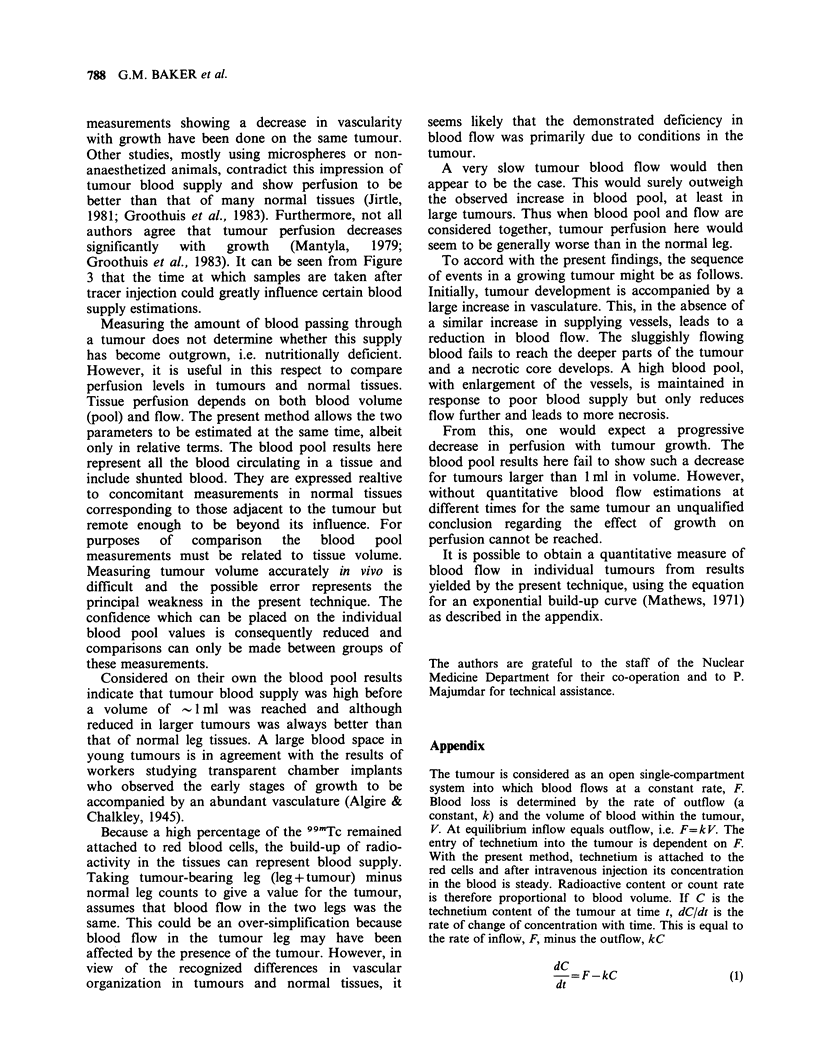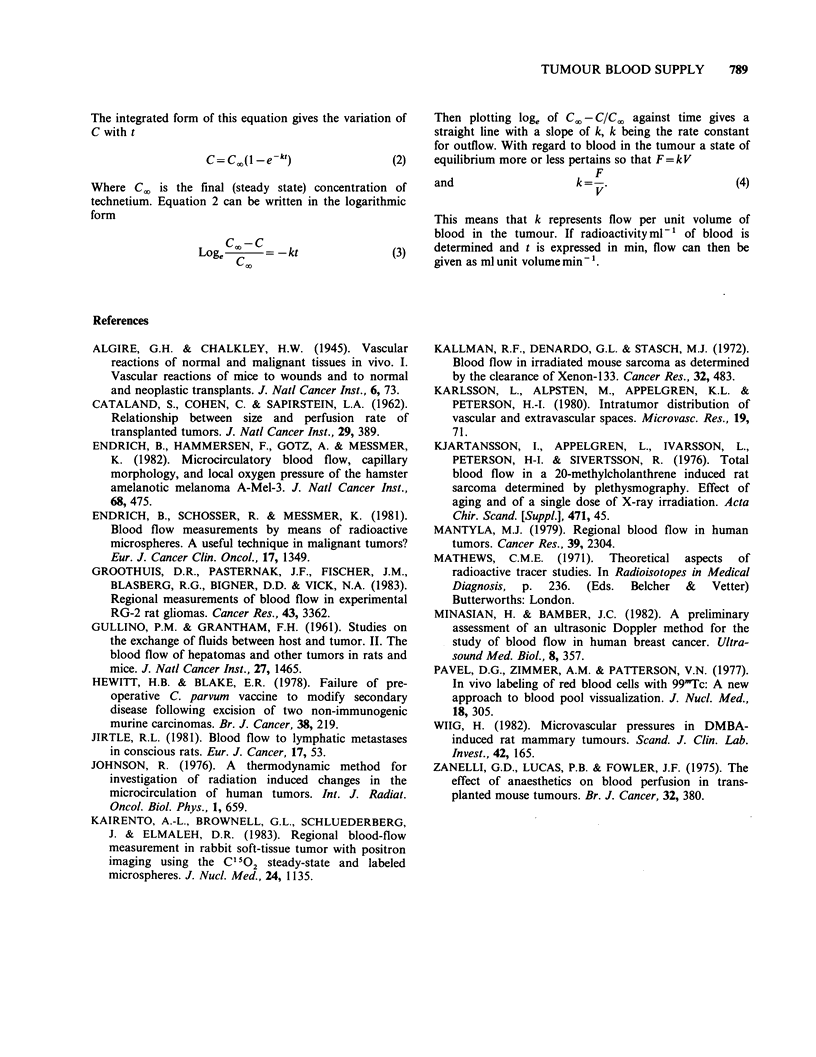# Measurement of blood supply to murine tumours using in vivo red cell labelling and dynamic scintigraphy.

**DOI:** 10.1038/bjc.1985.122

**Published:** 1985-06

**Authors:** G. M. Baker, M. B. Clarke, W. F. Whimster

## Abstract

**Images:**


*Br. J. Cancer* (1985), **51**, 783–789

### Measurement of blood supply to murine tumours using *in vivo* red cell labelling and dynamic scintigraphy

G.M. Baker[1], M.B. Clarke[2] & W.F. Whimster[1]

[1]*Department of Morbid Anatomy, King's College School of Medicine and Dentistry, Denmark Hill, London SE5 8RX; and* [2]*Department of Medical Physics, King's College Hospital, Denmark Hill, London SE5 9RS, UK.*

**Summary** Blood pool and flow were studied in transplanted adenocarcinomas on the legs of mice. The animals' red blood cells were labelled *in vivo* by consecutive injections of a stannous compound and $^{99m}$Tc-pertechnetate. The distribution of radioactivity was then recorded continuously with a gamma camera. This method allows prolonged and repeated estimations of blood supply to undisturbed tumours in conscious mice.

It was found that in small tumours (under 1 ml) circulating blood pool was usually high, often 2 or 3 times that in normal leg tissues. In tumours bigger than 1 ml blood pool per unit volume tended to be lower but was still about 1.5 times the normal tissue level. This relatively large blood volume would seem to be outweighed by a very slow rate of flow. Even in the small tumours blood perfusion was greatly reduced compared to that in the normal leg. The blood pool results here provide no evidence that in tumours larger than 1 ml blood supply decreased progressively with growth.

Knowledge concerning the blood supply of tumours is essential not only for the understanding of tumour biology but also if such treatments as chemotherapy, radiotherapy and hyperthermia are to be administered to the best advantage. Up until now, the methods used to measure tumour perfusion have had serious drawbacks.

Histological and angiographic studies demonstrate the existence and patency of vessels rather than blood supply and are at best only semi-quantitative. The use of radioactive tracers allows the actual measurement of blood flow, and the uptake by tumours of intravenously injected $^{86}$Rb, $^{42}$K or labelled iodoantipyrine has been assessed (Gullino & Grantham, 1961; Groothuis *et al.*, 1983). In addition, blood volume has been estimated using $^{125}$I-labelled albumin or $^{59}$Fe-labelled red cells (Karlsson *et al.*, 1980). Since these methods, like those involving histology, depend on taking tissue samples the animals must be killed to obtain the results. This has two major disadvantages. Firstly, only one measurement can be made for each tumour. Secondly, as pointed out by Algire & Chalkley (1945), a variable amount of the blood in a tumour usually leaks out when the host is killed, making post-mortem estimations of blood pool subject to error. The use of labelled microspheres can overcome the second problem but selection of the correct size of sphere is crucial and made difficult by the inhomogeneity of tumour vasculature (Endrich *et al.*, 1981).

Ingenious *in vivo* methods of estimating tumour blood supply have been devised but are not free from criticism. Many of them including the tissue-isolated implants of Gullino & Grantham (1961), plethysmography (Kjartansson *et al.*, 1976) or measurements of perfusion pressure (Wiig, 1982) require the animals to be anaesthetized. Anaesthetics are known to affect tumour blood flow differentially (Zanelli *et al.*, 1975) and their use produces questionable results. Red cell velocity has been measured in tumours growing in transparent chambers in the cheek pouches of non-anaesthetized hamsters (Endrich *et al.*, 1982). However, such tumours are not growing in a completely normal situation and the period of observation is curtailed by the limitations to growth imposed by the chamber. Another method which has been used on conscious animals involves the clearance of $^{133}$Xe from tumours (Kallman *et al.*, 1972). However, only regional blood flow has been measured because the isotope was introduced locally into the tumour and/or the area "seen" by the counter was limited. With methods involving heat transfer (Johnson, 1976) there is the danger of temperature-induced changes in the circulation. Laser and ultrasound Doppler techniques (Minasian & Bamber, 1982) are non-invasive but again only give information on local blood flow.

Recently, new methods utilizing short-lived isotopes, such as $^{15}O_2$, and computerized tomography have been applied to the measurement of regional blood flow in tumours. Most of the work has been done on human tumours but Kairento *et al.* (1983) used positron emission tomography to study tumour blood flow in rabbits. These

Correspondence: G.M. Baker.
Received 20 September 1984; and in revised form 14 February 1985

© The Macmillan Press Ltd., 1985

techniques have great protential, particularly when the at present rather poor spatial resolution has been improved.

The technique described in the present paper avoids all of the problems referred to above. Total blood supply to an undisturbed tumour can be assessed both over a period, and repeatedly, in a conscious animal.

### Materials and methods

### *Animals and tumour line*

The experimental animals were from a colony of WHT/Ht mice bred by brother-sister mating in the animal house at King's College Hospital Medical School. These mice were brought to King's in 1977 by H.B. Hewitt from the The Gray Laboratory, Mount Vernon Hospital (Northwood, Middlesex, UK.). The tumour line derives from an adenocarcinoma (probably mammary) which arose spontaneously in these mice (Hewitt & Blake, 1978). It has subsequently been maintained by serial subcutaneous transplantation. This was done in the present experiments by mashing an aseptically excised tumour in sterile 0.9% sodium chloride solution in a glass homogenizer and injecting 0.01–0.02 ml of the stirred suspension subcutaneously into the left calf of the mouse.

### *Tumour measurement*

Transplants were measured at intervals to establish that they were growing satisfactorily. They were also measured immediately before each blood pool estimation (Table I). Three orthotomic diameters were found to the nearest 0.5 mm using callipers. Tumour volume was then calculated from the formula for the volume of a sphere

$$V=\frac{4\pi r^3}{3}=\frac{\pi d^3}{6}=0.524 d_1 d_2 d_3.$$

The error involved in this was investigated in the following way. After diameter measurements 21 tumours were dissected out and their volumes found by water displacement. They ranged from 0.1 to 4.1 ml. Their volumes were also calculated as described above. Calculated and measured volumes were then compared. The mean difference was 12.8% (s.d. 7.8). Volumes estimated from calliper measurements were usually too high for tumours under 1 ml in size and too low for larger tumours.

### *Red cell labelling*

For blood pool measurement the red cells were labelled *in vivo* with $^{99m}$Technetium. This is accomplished by injecting a stannous compound to load the red cells with stannous ion, before an injection of $^{99m}$Tc-pertechnetate. Under these conditions $^{99m}$Tc is reduced within the stannous loaded cells and bound. Labelling is at its highest 15–30 min after pertechnetate injection but is still almost as high after 1 h (Pavel *et al.*, 1977).

Each mouse was injected with Amerscan (Amersham International plc, Amersham, Buckinghamshire, UK), a mixture of 4 mg stannous fluoride and 6.8 mg sodium medronate which was reconstituted with 0.9% NaCl. These injections consisted of 3.24 $\mu$g in 0.03 ml and were usually given subcutaneously in the tail. Subcutaneous injections into the right hind limb or i.v. injections into the tail were also tried but did not affect the outcome. Between 0.5 and 2.5 h later a second injection of $\sim$37 MBq (1 mCi) of $^{99m}$Tc-pertechnetate in 0.05–0.1 ml of 0.9% NaCl was given either s.c. or into a vein in the tail.

Blood samples were taken on several occasions in the course of the experiments from 6 mice and the efficiency of red cell labelling checked. These samples were taken between 10 and 60 min after pertechnetate injection, with the stannous injections 46–91 min before those of pertechnetate. Each heparinized blood sample was diluted with about 4 ml of 0.9% NaCl and spun down. Cells and supernatant were then counted separately with a NaI scintillation counter. The mean red cell count was 95.3% (s.d. 2.7) of the total blood count.

### *Blood pool estimation*

Shortly before pertechnetate injection the mouse was immobilized on a purpose-made jig, the construction of which is shown in Figure 1. The mouse was confined in a crouched position by means of a moulded Cabulite cover screwed to the base. The hind legs were extended laterally and fastened at the ankles by wire staples pushed into pieces of cork. This arrangement did not cut off blood circulation to the feet. Care was taken to position the spread-eagled mouse symmetrically because it was important that equal volumes of the two hind legs should be counted. If a mouse moved during counting it was repositioned and previous results disregarded. The injection site on the tail was shielded below by a sheet of 6 mm lead and above by a U-shaped lead cover. Time was allowed for the mouse to settle down before proceeding.

The jig with a mouse in position was placed on the collimator of an Elscint CE1-7 gamma camera (Elscint Ltd., Berinsfield, Oxfordshire, UK). The low energy, parallel-holed collimator was covered with a plastic-backed paper sheet and the jig was placed directly on this. $^{99m}$Tc-pertechnetate was then injected into the tail. The distribution of radio-

**Figure 1** Construction of jig used to immobilize the mouse for $^{99m}$Tc-pertechnetate injection and counting with a gamma camera.

activity (labelled red cells) in the mouse was displayed as a scintigram on a television screen. During counting, continuous series of images were stored on floppy discs. Each image represented a counting period of 0.5, 1, 2 or 6 sec, the duration increasing with time after injection. Three series of images were recorded for each mouse. One series extended from just before pertechnetate injecion to 7 or 10 min after (Period 1), and the other two from about 15–20 and 25–30 min post-injection (Periods 2 and 3). A $64 \times 64$ matrix was used for all images, each element being $3 \times 3\,mm^2$. In tumour-bearing mice the whole procedure was carried out on three occasions during tumour growth.

The records on floopy discs were subsequently analysed using a computer (A.D.A.C. System I, Analog Data Associates Corp., California, USA). The images were displayed on a television screen and areas of interest outlined by means of a light pen (Figure 2). In Figure 2, area A contains the normal (right) hind leg and area B the tumour-bearing leg. Total counts for these areas were then given per image by the computer. For each image, area A (normal leg) counts were subtracted from area B (tumour-bearing leg) counts to give a value for the tumour. Normal leg and tumour counts

**Figure 2** Scintigram as shown on TV screen of computer. Two areas of interest have been outlined, "A" containing the normal leg and "B" the tumour-bearing leg. [Because the image is from the below, the tumour-bearing leg is on the right].

were then averaged over 5 min periods of counting. A value for non-tumour-bearing leg volume had been found previously by water displacement. The mean volume of 21 normal right legs was 1.5 ml (s.d. 0.2). Two-thirds of the normal leg count therefore represented counts $ml^{-1}$. Since tumour volume was known tumour counts $ml^{-1}$ could be calculated. For each period of counting these results were used to find the ratio

$$\frac{\text{Specific blood volume in tumour} \times 100}{\text{Specific blood volume in normal tissues}}$$

giving a value for specific tumour blood pool (i.e. per ml) as a percentage of that of normal tissues. Hereafter, these quantities are referred to simply as blood pool.

Blood pool was measured in both normal and tumour-bearing legs. In mice with tumours it was measured on three occasions separated by intervals of 3–12 days. Relative blood flow in the two legs was estimated at the same times. Statistical significance between sets of results was determined using the Mann-Whitney $U$ test.

### Results

### *Normal leg blood pool*

A comparison of blood pool values from right and left hind legs of non-tumour-bearing mice provided a measure of error resulting from the technique. Leg counts from 13 normal mice were averaged over each of three 5 min periods (the last 5 min of Period

1 and the whole of Periods 2 and 3). In three cases the mouse pulled one leg free during counting so that it was no longer correctly placed on the jig. The movement had to be rectified and counts obtained before this were disregarded. The corresponding means for right and left legs were then compared. With one exception, the difference between the two legs was within 11%, mean 3.7% (s.d. 3.0). The exceptional case showed a difference of about 25%. This was probably due to movement, which was found to produce differences of the same order. Ratios of left and right leg counts show a mean which is not significantly different from 1 (mean = 1.01; $P = 0.12$).

*Tumour blood pool*

Each of 15 tumour-bearing mice was injected and counted on three occasions during the growth of its tumour. Pertechnetate injection was s.c. except where indicated in Table I. Change in normal leg count rate was found to be small over the third period of counting. For 71 normal-leg measurements the average difference between the means of five counts at the beginning of Period 3 and five at the end was 4% (s.d. 3.1). Thus radioactivity in the blood was fairly stable for Period 3, and count rates for normal and tumour-bearing legs were averaged over the 5 min of this period. When pertechnetate injection was intravenous counts were averaged over Periods 2 and 3 because in both of these variation between beginning and end of period count rates was within 10%. Average counts $ml^{-1}$ were then used to calculate tumour blood pool as a percentage of normal-leg-tissue blood pool for the three measurements of each tumour. These results are given in Table I.

Tumour blood pool was very variable ranging from 67–439%. The average measurement was 183% (s.d. 82). If the tumours are grouped according to age, percentage blood pool averages (±s.e) for 14–22, 24–29 and 33–37 day groups respectively are 222±22, 159±10 and 160±24. At 95% confidence level there is a aignificant difference between measurements from the youngest group and the rest but not between those from the two older groups. Another way of considering the results is according to tumour size. Taking four groups with tumour volumes of under 1 ml, 1–2 ml, 2–3 ml and 3–4 ml, percentage blood pool measurements average (±s.e.) 250±25, 156±7, 170±27 and 128±14 respectively. Blood pool in the under 1 ml group is significantly high compared with the others ($P < 0.025$), but differences between the three groups of larger tumours are not significant ($P < 0.25$).

Thus, tumours under 1 ml in size tended to have a blood pool per unit volume higher than that of larger tumours, often 2–3 times that of normal limb tissues. Blood pool in these small tumours will have been under- rather than over-estimated because of the likelihood that their volume measurements were too high. By the time a tumour volume of 1 ml was reached, average blood pool was reduced but was still ~1.5 times that of the normal leg tissues. Further 2- to 3-fold increase in tumour size did not

**Table I** Blood pool in tumours measured at three stages during their growth

| | *First measurement* | | | *Second measurement* | | | *Third measurement* | | |
|---|---|---|---|---|---|---|---|---|---|
| *Tumour no.* | *Age (days)* | *Size (ml)* | *Blood pool*[a] (%) | *Age (days)* | *Size (ml)* | *Blood pool* (%) | *Age (days)* | *Size (ml)* | *Blood pool* (%) |
| 1 | 14 | 0.2 | 374 | 22 | 1.2 | 151 | 26 | 2.0 | 161 |
| 2 | 14 | 0.3 | 223 | 22 | 1.4 | 211 | 26 | 2.6 | 160 |
| 3 | 14 | 0.5 | 337 | 26 | 2.6 | 102 | 29 | 3.5 | 124 |
| 4 | 17 | 0.9 | 172 | 24 | 1.6 | 150 | 34 | 3.6 | 168 |
| 5 | 17 | 0.5 | 272 | 24 | 1.2 | 170 | 34 | 3.0 | 112 |
| 6 | 17 | 0.2 | 419 | 24 | 0.7 | 261 | 34 | 2.4 | 105 |
| 7 | 20 | 0.3 | 134 | 28 | 1.1 | 154 | 34 | 2.2 | 131 |
| 8 | 20 | 0.3 | 126 | 28 | 1.1 | 182 | 34 | 2.0 | 67 |
| 9 | 20 | 0.5 | 174[b] | 28 | 1.7 | 127 | 37 | 3.9 | 81 |
| 10 | 22 | 0.8 | 218 | 27 | 1.5 | 170 | 35 | 2.7 | 194 |
| 11 | 22 | 1.5 | 155 | 27 | 2.1 | 150 | 35 | 3.6 | 118[b] |
| 12 | 22 | 1.5 | 112[b] | 27 | 2.3 | 179 | 35 | 2.8 | 174 |
| 13 | 22 | 1.1 | 158 | 27 | 1.8 | 133 | 36 | 3.0 | 166 |
| 14 | 22 | 0.4 | 240 | 33 | 1.2 | 167 | 36 | 1.6 | 146 |
| 15 | 22 | 0.6 | 299 | 33 | 2.6 | 177 | 36 | 2.5 | 439 |

[a]Mean tumour count $ml^{-1}$ as a percentage of mean normal leg count $ml^{-1}$.
[b]$^{99m}Tc$-Pertechnetate injection intravenous.

produce any regular, statistically significant change in blood pool.

*Dissimilarity between normal tissue and tumour blood flow*

Whereas counts at equilibrium gave a measure of blood pool, the initial rate of increase in counts was an indication of relative blood flow. For 43 out of the 45 measurements, data was available to show that 2 min after the start of counting (~1.5 min after injection) the build-up of radioactivity was more gradual in the legs carrying tumours. Count rate at this time, expressed as a percentage of the final rate (mean count for Period 3) was always lower than in the normal leg. The situation is seen best after intravenous pertechnetate injection, when the technetium level in the blood was steady. Thus for the three mice injected i.v. (Table I) count rates for normal leg, tumour-bearing leg and tumour (difference between tumour-bearing and normal legs) were plotted against time. This is shown in Figure 3, where each point represents the average of results from the three mice. In normal legs, counts rose sharply over approximately the first 10 sec and then slowly until a maximum was reached at about 2 min post-injection. For the rest of Period 1 count rate in the normal leg decreased at ~3% $min^{-1}$. In contrast, tumour counts rose less sharply at the beginning and continued to rise longer. Two min after injection tumour count rate was still rising by ~14% $min^{-1}$, and a maximum was not reached until 3.5–6 min post-injection.

During Periods 2 and 3 (~15–19.5 and 25–29.5 min after injection respectively) count rates for both tumours and normal legs showed a steady fall of 1 or 2% $min^{-1}$. Radioactive decay of the isotope would account for a fall in counts of ~0.2% $min^{-1}$. Loss of the small percentage of free $^{99m}$Tc from the blood, due to accumulation in organs such as the thyroid, could also cause a decrease in limb and tumour counts.

From the difference between the curves for Period 1 in Figure 3 it appears that the flow of blood through the tumours was much slower than that through the normal leg tissues.

### Discussion

Many of the results from methods described in the introduction have led to the idea that in time tumours outgrow their blood supply, which is then poor compared to that of normal tissues. It has been shown that vascularity and blood flow in tumours are inversely related to size and age (Cataland *et al.*, 1962; Karlsson *et al.*, 1980; Gullino & Grantham, 1961). Such results would be promoted by the study of tumours growing in unnatural circumstances or of post-mortem material, and by the use of anaesthetics. Few of the

**Figure 3** Count rates for tumour-bearing legs (■), normal legs (□) and tumours (● = difference between counts for tumour-bearing and normal legs) plotted over the 3 periods of counting following intravenous pertechnetate injection. Points are means of results from 3 mice.

measurements showing a decrease in vascularity with growth have been done on the same tumour. Other studies, mostly using microspheres or non-anaesthetized animals, contradict this impression of tumour blood supply and show perfusion to be better than that of many normal tissues (Jirtle, 1981; Groothuis *et al.*, 1983). Furthermore, not all authors agree that tumour perfusion decreases significantly with growth (Mantyla, 1979; Groothuis *et al.*, 1983). It can be seen from Figure 3 that the time at which samples are taken after tracer injection could greatly influence certain blood supply estimations.

Measuring the amount of blood passing through a tumour does not determine whether this supply has become outgrown, i.e. nutritionally deficient. However, it is useful in this respect to compare perfusion levels in tumours and normal tissues. Tissue perfusion depends on both blood volume (pool) and flow. The present method allows the two parameters to be estimated at the same time, albeit only in relative terms. The blood pool results here represent all the blood circulating in a tissue and include shunted blood. They are expressed realtive to concomitant measurements in normal tissues corresponding to those adjacent to the tumour but remote enough to be beyond its influence. For purposes of comparison the blood pool measurements must be related to tissue volume. Measuring tumour volume accurately *in vivo* is difficult and the possible error represents the principal weakness in the present technique. The confidence which can be placed on the individual blood pool values is consequently reduced and comparisons can only be made between groups of these measurements.

Considered on their own the blood pool results indicate that tumour blood supply was high before a volume of $\sim$1 ml was reached and although reduced in larger tumours was always better than that of normal leg tissues. A large blood space in young tumours is in agreement with the results of workers studying transparent chamber implants who observed the early stages of growth to be accompanied by an abundant vasculature (Algire & Chalkley, 1945).

Because a high percentage of the $^{99m}$Tc remained attached to red blood cells, the build-up of radioactivity in the tissues can represent blood supply. Taking tumour-bearing leg (leg+tumour) minus normal leg counts to give a value for the tumour, assumes that blood flow in the two legs was the same. This could be an over-simplification because blood flow in the tumour leg may have been affected by the presence of the tumour. However, in view of the recognized differences in vascular organization in tumours and normal tissues, it seems likely that the demonstrated deficiency in blood flow was primarily due to conditions in the tumour.

A very slow tumour blood flow would then appear to be the case. This would surely outweigh the observed increase in blood pool, at least in large tumours. Thus when blood pool and flow are considered together, tumour perfusion here would seem to be generally worse than in the normal leg.

To accord with the present findings, the sequence of events in a growing tumour might be as follows. Initially, tumour development is accompanied by a large increase in vasculature. This, in the absence of a similar increase in supplying vessels, leads to a reduction in blood flow. The sluggishly flowing blood fails to reach the deeper parts of the tumour and a necrotic core develops. A high blood pool, with enlargement of the vessels, is maintained in response to poor blood supply but only reduces flow further and leads to more necrosis.

From this, one would expect a progressive decrease in perfusion with tumour growth. The blood pool results here fail to show such a decrease for tumours larger than 1 ml in volume. However, without quantitative blood flow estimations at different times for the same tumour an unqualified conclusion regarding the effect of growth on perfusion cannot be reached.

It is possible to obtain a quantitative measure of blood flow in individual tumours from results yielded by the present technique, using the equation for an exponential build-up curve (Mathews, 1971) as described in the appendix.

The authors are grateful to the staff of the Nuclear Medicine Department for their co-operation and to P. Majumdar for technical assistance.

### Appendix

The tumour is considered as an open single-compartment system into which blood flows at a constant rate, $F$. Blood loss is determined by the rate of outflow (a constant, $k$) and the volume of blood within the tumour, $V$. At equilibrium inflow equals outflow, i.e. $F=kV$. The entry of technetium into the tumour is dependent on $F$. With the present method, technetium is attached to the red cells and after intravenous injection its concentration in the blood is steady. Radioactive content or count rate is therefore proportional to blood volume. If $C$ is the technetium content of the tumour at time $t$, $dC/dt$ is the rate of change of concentration with time. This is equal to the rate of inflow, $F$, minus the outflow, $kC$

$$\frac{dC}{dt}=F-kC \qquad (1)$$

The integrated form of this equation gives the variation of $C$ with $t$

$$C = C_\infty(1 - e^{-kt}) \quad (2)$$

Where $C_\infty$ is the final (steady state) concentration of technetium. Equation 2 can be written in the logarithmic form

$$\text{Log}_e \frac{C_\infty - C}{C_\infty} = -kt \quad (3)$$

Then plotting $\log_e$ of $C_\infty - C/C_\infty$ against time gives a straight line with a slope of $k$, $k$ being the rate constant for outflow. With regard to blood in the tumour a state of equilibrium more or less pertains so that $F = kV$

and $$k = \frac{F}{V}. \quad (4)$$

This means that $k$ represents flow per unit volume of blood in the tumour. If radioactivity $\text{ml}^{-1}$ of blood is determined and $t$ is expressed in min, flow can then be given as ml unit volume $\text{min}^{-1}$.

### References

ALGIRE, G.H. & CHALKLEY, H.W. (1945). Vascular reactions of normal and malignant tissues in vivo. I. Vascular reactions of mice to wounds and to normal and neoplastic transplants. *J. Natl Cancer Inst.*, **6**, 73.

CATALAND, S., COHEN, C. & SAPIRSTEIN, L.A. (1962). Relationship between size and perfusion rate of transplanted tumors. *J. Natl Cancer Inst.*, **29**, 389.

ENDRICH, B., HAMMERSEN, F., GOTZ, A. & MESSMER, K. (1982). Microcirculatory blood flow, capillary morphology, and local oxygen pressure of the hamster amelanotic melanoma A-Mel-3. *J. Natl Cancer Inst.*, **68**, 475.

ENDRICH, B., SCHOSSER, R. & MESSMER, K. (1981). Blood flow measurements by means of radioactive microspheres. A useful technique in malignant tumors? *Eur. J. Cancer Clin. Oncol.*, **17**, 1349.

GROOTHUIS, D.R., PASTERNAK, J.F., FISCHER, J.M., BLASBERG, R.G., BIGNER, D.D. & VICK, N.A. (1983). Regional measurements of blood flow in experimental RG-2 rat gliomas. *Cancer Res.*, **43**, 3362.

GULLINO, P.M. & GRANTHAM, F.H. (1961). Studies on the exchange of fluids between host and tumor. II. The blood flow of hepatomas and other tumors in rats and mice. *J. Natl Cancer Inst.*, **27**, 1465.

HEWITT, H.B. & BLAKE, E.R. (1978). Failure of pre-operative *C. parvum* vaccine to modify secondary disease following excision of two non-immunogenic murine carcinomas. *Br. J. Cancer*, **38**, 219.

JIRTLE, R.L. (1981). Blood flow to lymphatic metastases in conscious rats. *Eur. J. Cancer*, **17**, 53.

JOHNSON, R. (1976). A thermodynamic method for investigation of radiation induced changes in the microcirculation of human tumors. *Int. J. Radiat. Oncol. Biol. Phys.*, **1**, 659.

KAIRENTO, A.-L., BROWNELL, G.L., SCHLUEDERBERG, J. & ELMALEH, D.R. (1983). Regional blood-flow measurement in rabbit soft-tissue tumor with positron imaging using the $C^{15}O_2$ steady-state and labeled microspheres. *J. Nucl. Med.*, **24**, 1135.

KALLMAN, R.F., DENARDO, G.L. & STASCH, M.J. (1972). Blood flow in irradiated mouse sarcoma as determined by the clearance of Xenon-133. *Cancer Res.*, **32**, 483.

KARLSSON, L., ALPSTEN, M., APPELGREN, K.L. & PETERSON, H.-I. (1980). Intratumor distribution of vascular and extravascular spaces. *Microvasc. Res.*, **19**, 71.

KJARTANSSON, I., APPELGREN, L., IVARSSON, L., PETERSON, H-I. & SIVERTSSON, R. (1976). Total blood flow in a 20-methylcholanthrene induced rat sarcoma determined by plethysmography. Effect of aging and of a single dose of X-ray irradiation. *Acta Chir. Scand.* [*Suppl.*], **471**, 45.

MANTYLA, M.J. (1979). Regional blood flow in human tumors. *Cancer Res.*, **39**, 2304.

MATHEWS, C.M.E. (1971). Theoretical aspects of radioactive tracer studies. In *Radioisotopes in Medical Diagnosis*, p. 236. (Eds. Belcher & Vetter) Butterworths: London.

MINASIAN, H. & BAMBER, J.C. (1982). A preliminary assessment of an ultrasonic Doppler method for the study of blood flow in human breast cancer. *Ultrasound Med. Biol.*, **8**, 357.

PAVEL, D.G., ZIMMER, A.M. & PATTERSON, V.N. (1977). In vivo labeling of red blood cells with $99^mTc$: A new approach to blood pool vissualization. *J. Nucl. Med.*, **18**, 305.

WIIG, H. (1982). Microvascular pressures in DMBA-induced rat mammary tumours. *Scand. J. Clin. Lab. Invest.*, **42**, 165.

ZANELLI, G.D., LUCAS, P.B. & FOWLER, J.F. (1975). The effect of anaesthetics on blood perfusion in transplanted mouse tumours. *Br. J. Cancer*, **32**, 380.